# Energy, environment, and economy implications of electrifying minibus taxis in African cities

**DOI:** 10.1038/s41598-026-45790-w

**Published:** 2026-03-30

**Authors:** Jérémy Dumoulin, Alejandro Pena-Bello, Noémie Jeannin, Christophe Ballif, Nicolas Wyrsch

**Affiliations:** Photovoltaics and thin film electronics laboratory (PV-LAB), École Polytechnique Fédérale de Lausanne (EPFL), Institute of Electrical and Microengineering (IEM), Neuchâtel, Switzerland

**Keywords:** Energy science and technology, Engineering

## Abstract

The electrification of minibus taxis in Africa is envisioned to play an important role in decarbonising transportation, reducing urban air pollution, and generating economic savings. However, comprehensive quantifications of these implications are scarce. This study introduces a modelling approach based on readily available data to simulate minibus operation and evaluate the key energy, environmental, and economic implications of such an electrification. Applying our model to nine diverse African cities, we find that electrifying minibuses could prevent the emission of 4.3 to 19.2 tCO$$_2$$ annually per vehicle, based on current electricity mixes, while saving minibus owner-operators US$1.2k to US$14.0k in annual fuel costs. Using a relative exposure index for minibus-related air pollution, we also identify that approximately 23 million people across these cities could benefit from improved air quality. Nevertheless, substantial variations in the charging demand–both per vehicle and aggregated per city–are observed, emphasising the critical importance of energy planning and tailored electrification strategies. This research provides insights for policymakers and planners, and offers a transparent and replicable framework for assessing the impacts of public transport electrification across diverse locations.

## Introduction

Vehicle electrification, coupled with the rapid development of renewable electricity generation, presents a transformative opportunity to decarbonise Africa’s road-dependent transport sector^1–3^. It also offers many other benefits, such as economic savings^4^ and reduced air pollution^5^, potentially preventing the premature death of several hundred thousand people across the continent annually^6^. Although the electrification is in its early stages, the momentum towards rapid adoption of electric vehicles is gaining traction. Indeed, a growing number of African nations are encouraging their adoption by implementing ambitious goals and measures, such as tight regulations on used-vehicle imports^7–9^. Nevertheless, research on the major impacts and most effective strategies for widespread electric vehicle adoption in Africa remains notably scarce. Understanding the implications of this transition is crucial for many aspects, such as infrastructure planning, grid management, electric vehicle battery sizing, economic prospects, and policy formulation^10^.

To effectively address this topic, research must delve into the unique African mobility landscape, characterised by diverse and spatially variable transport modes and habits^11,12^. This diversity is particularly pronounced in the passenger transport sector. Notably, since the decline of formal public transport in the 1990s following structural adjustments in World Bank policies^13,14^, informal public transport (commonly known as paratransit) has become a cornerstone of daily mobility. In numerous urban and semi-urban areas across the continent, it has emerged as the predominant mode of transportation^15–17^, offering flexible and efficient transport to millions of individuals with minimal public investment^18^. The paratransit sector encompasses a wide range of vehicle types, including motorcycles, cars, auto-rickshaws, and, most prominently, minibus taxis^1^. Their operating patterns vary widely: in some contexts they resemble formal public transport with fixed routes and timetables, while elsewhere they follow flexible schedules with departure times largely determined by passenger demand^1,15,19^. These minibuses, typically second-hand vehicles with an average age of around 15 years^13^, should eventually transition to electric models. Given the widespread presence of paratransit in Africa, such a transition could yield substantial benefits and fundamentally transform the continent’s transportation and energy sectors. These transformations are likely to occur primarily in cities, where paratransit services are mostly concentrated^12,20,21^ and access to electricity is higher than in rural areas^22^. Moreover, with Africa experiencing unprecedented urbanisation, studying electric minibus adoption at the city level helps anticipate future energy demand and informs potential scaling.

Research on paratransit electrification in Africa is still in its early stages but is gaining traction as cities explore low-carbon mobility options. Most existing studies focus on vehicle-level energy use, greenhouse-gas reduction potential, and operational aspects of electric minibuses^14,23–30^. However, several important knowledge gaps remain. First, comprehensive assessments across multiple locations are scarce. The majority of empirical and modelling studies have been conducted in South Africa, with few multi-city or multi-country analyses to provide generalisable insights^14,31^. Consequently, findings from one context may not translate to others due to differences in local energy landscapes. For example, electricity access quality, electricity prices, and the carbon intensity of power generation vary widely across the continent^22^. One study of South Africa’s coal-dominated grid even suggested that EV deployment could paradoxically increase emissions if not paired with renewable energy sources^32^. To our knowledge, only Rix et al.^24^ have evaluated charging demand across multiple cities, yet without fully contextualising this demand within local energy landscapes and assess economic and environmental implications. Second, existing research often relies on data-intensive methods, such as detailed GPS tracking and micro-traffic simulations, to estimate energy requirements^23,27,29,30^. While these approaches provide detailed insights for specific contexts, they are difficult to replicate in data-scarce settings, limiting broader applicability across Africa. The scarcity of high-frequency mobility data is a well-known challenge^21^, emphasising the need for models that can operate with more accessible datasets, such as the widely available General Transit Feed Specification (GTFS)^33–35^. Third, the analysis of environmental and economic implications remains incomplete. While most studies focus on CO$_2$ emissions reduction^29,32^, other crucial implications such as economic savings for minibus owner-operators or air pollution reduction are underexplored^17,36^. Paratransit services, while easily accessible in urban areas^17^, expose a large portion of the population to the heavily polluting minibuses^36^. Addressing all these aspects is crucial to develop a comprehensive understanding of electric minibus deployment across Africa.

In this article, we aim to address the existing research gaps by estimating the main energy, economic, and environmental implications of electrifying minibus taxis in African cities. We present a modelling method that relies solely on easily accessible data (see “Methods” and Supplementary Fig. 1 for detailed modelling steps), applying it to nine major African cities: Abidjan, Accra, Alexandria, Bamako, Cairo, Freetown, Harare, Kampala, and Nairobi. These cities were selected to capture a broad diversity of urban contexts, ranging from large metropolitan areas (e.g., Cairo, Nairobi) to smaller but rapidly growing capitals (e.g., Bamako, Freetown). All nine cities rely extensively on minibuses for public transport, making them particularly relevant for assessing the implications of fleet electrification. The selection was further guided by the availability of data (see “Methods” for data sources) and by contrasting population densities along paratransit routes, which allow for an assessment of how population exposure to emissions from conventional minibuses varies across urban contexts. Our model centres on simulating minibus operation and electricity demand on a typical weekday, leveraging General Transit Feed Specification (GTFS) transport data made publicly available through the DigitalTransport4Africa initiative^35^. The model considers both individual vehicle and entire fleet perspectives. The projected electricity demand is juxtaposed against the current electricity consumption in the studied cities to provide insights into potential impacts on the local electricity systems. From an economic standpoint, we assess potential cost savings for minibus owner-operators resulting from fuel savings, accounting for the impact of existing diesel subsidies. From an environmental standpoint, our methodology estimates potential reductions in CO$_2$ emissions and exposure to traffic-related air pollution. By using georeferenced population data, we calculate the number of people exposed to air pollution from conventional minibuses at both city-wide and hectare-level resolutions, enabling the identification of air pollution exposure hotspots. Additionally, we discuss potential charging scenarios, including the feasibility of using locally installed photovoltaic energy to meet the charging needs. This multifaceted analysis provides a global overview along with city-specific insights for informed decision-making, supporting policymakers and planners in developing effective strategies for the widespread adoption of electric minibus taxis in Africa.

**Fig. 1:**
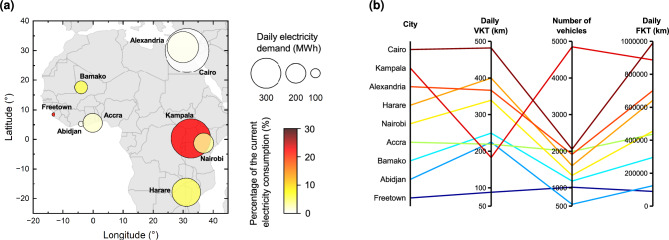
Total daily electricity demand for different cities and associated minibus operational characteristics. (**a**) Daily electricity demand for charging. The colour scale indicates the percentage (%) by which the additional electricity demand for charging minibuses increases the current city electricity consumption. (**b**) Main operational indicators derived from our model on a daily basis: VKT, number of vehicles on the different routes, FKT.

## Results

### Per-city electricity demand for charging

The additional electricity demand resulting from the potential 100 % electrification of minibus taxis in the nine studied cities is illustrated in Fig. 1a. The results are presented in two formats: the absolute daily charging demand (MWh) and the relative charging demand as a percentage (%) of the current city electricity consumption (for methodological details, see “Methods”). Furthermore, Fig. 1b depicts the key operational characteristics of the minibuses, highlighting important variations in minibus operation and the associated energy implications across the studied cities.

The daily charging demands vary across cities, ranging by nearly an order of magnitude from 38.8 MWh to 429.1 MWh. Specifically, Cairo, Kampala, and Alexandria exhibit charging demands exceeding 300 MWh, with 429.1 MWh for Cairo, 384.2 MWh for Kampala, and 303.9 MWh for Alexandria. In contrast, the demand for Freetown and Abidjan is only 38.8 MWh and 53.7 MWh, respectively. The observed differences are partly due to the population sizes in each city (Supplementary Table 3), estimated from a geographic analysis using gridded population data^37^ (see “Methods”). However, it is worthwhile to note that the electricity demand does not scale linearly with the population size, indicating that mobility habits and reliance on the paratransit system are also major drivers of the total electricity demand. For instance, although Kampala and Accra have comparable populations (with a difference of less than 10 %), the charging demand in Kampala is twice that of Accra. This suggests that the electricity demand cannot be determined solely based on the size of a city; instead, each paratransit system must be evaluated individually.

The operational characteristics presented in Fig. 1b reveal key differences that explain the variations in the total electricity demand. Firstly, the figure shows the daily fleet kilometres travelled (FKT), representing the total distance covered by the entire minibus fleet. Note that the FKT increases linearly with the daily charging demand because the electric energy consumption per kilometre and the charging efficiency are consistently applied to all vehicles across all cities (see “Methods”). Figure 1b also depicts the average daily distance travelled by minibus taxis, or vehicle kilometres travelled (VKT), along with the average number of vehicles on the road, emphasising differences in FKT origins. For instance, despite Kampala and Cairo having similar FKTs (990,191 km and 886,575 km, respectively), Kampala’s minibus taxi fleet is more than twice the size of Cairo’s (4850 minibuses and 2055 minibuses, respectively). Conversely, the VKT of Kampala is approximately half that of the Egyptian capital. These discrepancies may also impact energy disaggregation between vehicles based on the number of vehicles and the charging needs per vehicle. Hence, cities with higher VKTs will likely require minibuses equipped with larger battery capacities. Similarly, cities with larger vehicle fleets, such as Kampala and Cairo, will need more charging stations and grid upgrades to manage peak loads (see the Discussion section for further insights on potential smart charging scenarios to reduce these requirements).

The colour scale in Fig. 1a also reveals variations in the charging demand relative to the current city electricity consumption. While Cairo, Alexandria, and Abidjan exhibit low relative charging demands compared to their current electricity consumption (<1 %), Kampala and Freetown show notably higher values at 22.7 % and 21.8 %, respectively. In Kampala, this high percentage stems from the contrast between the substantial charging demand of 384.2 MWh and the city’s low per capita electricity consumption of only 96.0 kWh/year (see Supplementary Table 3). In Freetown, the current electricity consumption per capita is even lower (24.9 kWh/year), leading to a high percentage despite the charging demand being only 38.8 MWh. These findings underscore the critical need to integrate paratransit electrification into energy planning, particularly for cities like Kampala and Freetown, where the remarkably high charging demand compared to the current city electricity consumption could severely strain the local grid without adequate planning.

### Per-vehicle charging demand

**Fig. 2:**
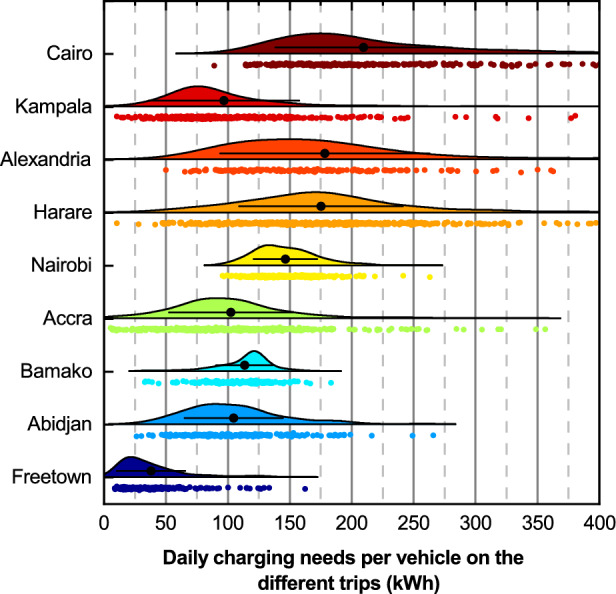
Distribution of the daily charging needs per minibus taxi on the different trips. The black dots and bars show the average and the standard deviation, respectively. Please note that each data point represents the charging needs per vehicle on the various trips. Therefore, the average value varies slightly from the charging needs one might anticipate based on the VKT illustrated in Fig. 1b, as the average value indicated here is not weighted by the number of vehicles in operation on each trip.

Figure 2 illustrates the daily charging demand for minibus taxis across trips, showing variations both between cities and among vehicles within the same city. Understanding these variations is crucial for developing effective electrification strategies, particularly regarding required battery capacities and charging strategies to meet the charging needs of all vehicles.

Examining the differences among cities, the results indicate an average charging demand (black dots in Fig. 2) ranging from 37.9 kWh in Freetown to 209.5 kWh in Cairo. Cities with shorter average trip distances and fewer trips per day, like Freetown, tend to have lower charging demands, while cities with longer trips and more trips per day like Cairo, exhibit higher charging demands. In Freetown and Kampala, the average charging demand is below 100 kWh, which can be adequately met with small electric minibus taxis currently available on the market (typically equipped with a battery capacity of around 70 kWh^38,39^). In contrast, vehicles operating in cities like Cairo, Harare, and Alexandria would require batteries with capacities exceeding 150 kWh to complete their day with a single charge. This capacity is more commonly found in large-capacity electric buses^40^, highlighting the necessity to explore smart operational strategies or smart charging solutions to reduce the required battery capacity if reliance on minibuses is to be maintained. Notably, examining the extreme values reveals that some minibuses in Cairo and Harare would need approximately 400 kWh per day, further emphasising the importance of implementing these advanced strategies in certain cities.

Figure 2 also demonstrates the marked differences in the charging demand among vehicles in certain locations. Cities like Nairobi and Bamako exhibit tight distributions around the mean, indicating consistent electricity demands across vehicles (standard deviations of 25.6 kWh and 23.0 kWh, with average charging demands of 146.5 kWh and 113.4 kWh, respectively). The two cities could benefit from uniform battery capacities and charging solutions, simplifying planning and potentially reducing purchase costs for minibus owner-operators managing multiple vehicles. In contrast, cities such as Cairo, Harare, and Alexandria show greater variation due to less uniformity in the number and length of daily trips per vehicle. For example, Alexandria has a standard deviation of 84.5 kWh, relative to an average charging demand of 178.3 kWh. Consequently, these cities will not only face a higher average charging demand but will also likely need a wider range of battery capacities and charging solutions to meet the more diverse charging needs.

### Changes in CO$_2$ emissions

**Fig. 3:**
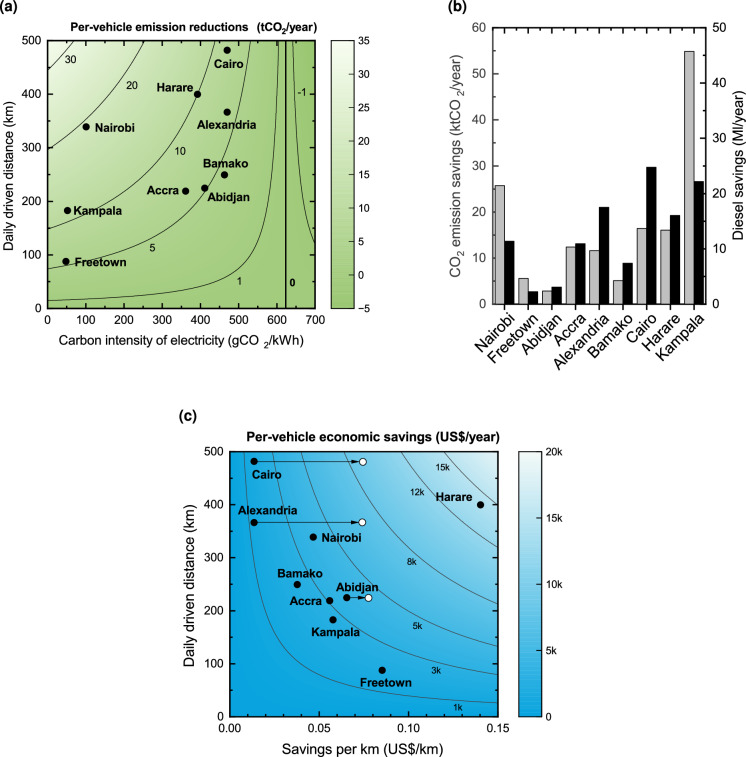
Yearly CO$$_2$$ savings and cost savings across cities associated with the adoption of electric minibuses. (**a**) Per-vehicle CO$$_2$$ savings as a function of the daily driven distance and the carbon intensity of electricity. (**b**) Total emission reductions per city (grey) and associated diesel savings (black). (**c**) Per-vehicle economic savings (in US$) for electric minibus owner-operators as a function of daily driven distance and savings per kilometre. The white dots indicate potential savings if diesel subsidies are removed.

The annual CO$_2$ reductions are examined for each city in Fig. 3, both in terms of the reduction per vehicle (Fig. 3a) and the total reduction per city, considering the entire vehicle fleet (Fig. 3b). Our findings indicate that in all cities, minibus taxi electrification would reduce CO$_2$ emissions. However, the extent of this reduction strongly depends on the city, influenced by local factors such as the carbon intensity of electricity and the number of kilometres driven by the vehicles.

Figure 3a depicts the average CO$_2$ savings per vehicle as a function of the two aforementioned factors. In all the studied cities, electric minibuses serve as a CO$_2$ sink compared to their thermal counterparts, with estimated CO$_2$ savings ranging from 4.3 to 19.2 tCO$_2$/year for Bamako and Nairobi, respectively. The contour lines in Fig. 3a demonstrate that CO$_2$ emissions decrease as long as the carbon intensity of electricity remains below 610 gCO$_2$/kWh - a threshold consistently met by the highly decarbonised electricity mix in most African countries^41^. For example, Sierra Leone and Uganda have a carbon intensity of electricity below 100 gCO$_2$/kWh. Interestingly, although Egypt’s carbon intensity of electricity is 470 gCO$_2$/kWh, the CO$_2$ savings for Cairo (8.0 tCO$_2$/year) and Alexandria (6.1 tCO$_2$/year) surpass those for Freetown (5.5 tCO$_2$/year). This is due to the longer average daily driven distances in these Egyptian cities. In Nairobi, the reduction reaches 19.2 tCO$_2$/year due to the combination of a low-carbon electricity mix (101 gCO$_2$/kWh) and a high average VKT (339 km). The potential for CO$_2$ savings would be even greater if the electricity mix were 100 % decarbonised, potentially increasing savings to a range of 5.9 to 32.5 tCO$_2$/year, for Freetown and Cairo, respectively.

As shown in Fig. 3b, electrifying minibus taxis could lead to total savings of 2.8-54.8 ktCO$_2$/year and 2.2-24.7 Ml/year of diesel, depending on the city. Overall, when aggregating the results over all cities, this corresponds to a reduction of 150.6 ktCO$_2$/year and 115.5 Ml/year. Considering the current emissions per capita in Africa, averaging around 0.9 tCO$_2$/year^2^, this indicates that the electrification of minibus taxis alone would save the equivalent of emissions from more than 160,000 people. It is also important to note that the largest CO$_2$ savings (Kampala) do not correspond to the largest diesel savings (Cairo), highlighting the potential benefit of further decarbonising the electricity mix. With a 100 % decarbonised electricity mix in Cairo, the total reduction would range from 6.0 ktCO$_2$/year (Freetown) to 66.8 ktCO$_2$/year (Cairo). The implications extend beyond these individual cities: if similar savings were projected to other cities, widespread adoption of electric minibuses could considerably reduce urban CO$_2$ emissions across the entire continent.

### Economic benefits

**Fig. 4:**
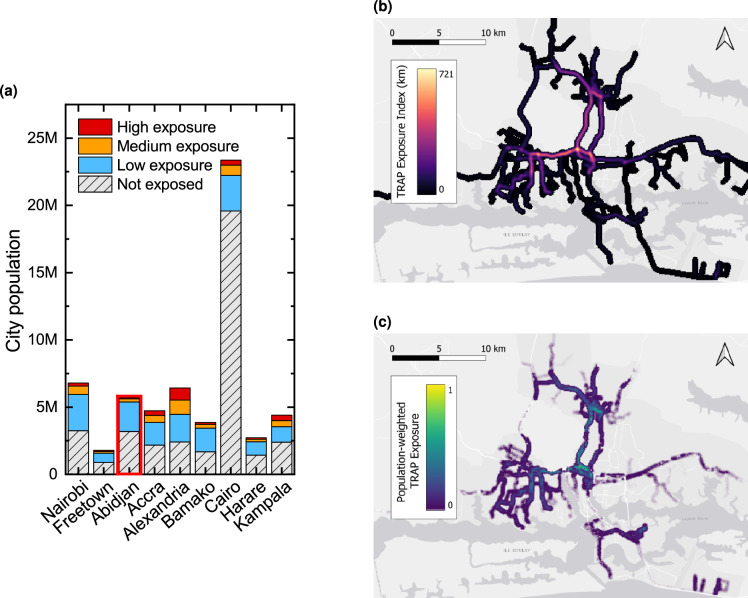
Assessment of the population’s exposure to traffic-related air pollution emitted by non-electric minibus taxis, with a focus on the city of Abidjan. (**a**) Total population exposed in the various cities and associated level of exposure based on the TRAP exposure index. (**b**) Map of the TRAP exposure index in Abidjan. (**c**) Map of population-weighted TRAP exposure index showing exposure hotspots.

The transition from thermal to electric vehicles presents an economic opportunity for minibus owner-operators due to potential fuel savings. These savings depend on two primary factors: the total distance travelled and the cost savings per kilometre (see Fig. 3c). The latter is notably high in many African countries due to relatively low household electricity prices compared to diesel fuel prices in most locations^42^ (see the Supplementary Information for a breakdown of fuel and electricity prices for each country).

Figure 3c indicates that switching to electric minibuses could lead to fuel savings in all studied cities. Harare demonstrates the highest potential savings. In this city, the average daily distance covered by minibuses is 399 kilometres (see Fig. 1). Operating a diesel-powered minibus over this distance incurs a cost of approximately US$67 per day, based on a diesel price of US$1.68 per litre in Zimbabwe. In contrast, an electric minibus can cover the same distance for only US$11, given a household electricity price of US$0.064 per kWh in Zimbabwe. Assuming 250 working days per year, the potential annual fuel savings for Harare amount to US$14.0k. In Accra, Abidjan, and Nairobi, the annual savings range from US$3.0-5.0k, while in other studied cities, they fall between US$1.0-3.0k.

These fuel savings could offset the purchase price of an electric minibus, especially as electric vehicle prices approach parity with their thermal counterparts^43^, with some Asian automobile manufacturers already offering small electric minibuses at comparable prices to thermal models^44^. A full total cost of ownership assessment lies beyond the scope of this study, as it would require detailed scenarios for specific vehicle models and charging strategies. Nonetheless, our results indicate that the overall operating costs of electric minibuses may be lower than those of fossil-fuel vehicles, since cost components other than fuel and purchase price (such as maintenance, staffing, and mid-life refurbishments) are expected to be similar or lower for electric minibuses^45^. A remaining uncertainty concerns the cost of the charging infrastructure, should this investment fall directly on operators. However, there remains considerable margin: fuel savings persist provided that charging costs stay below certain thresholds, up to 1.4 times the household electricity price in Bamako and up to 6 times in Harare. Moreover, large owner-operators might also secure lower electricity prices through bulk purchasing.

It is also crucial to note that numerous African countries implement substantial financial subsidies for diesel fuel. Figure 3c shows the economic savings without subsidies for Cairo, Alexandria, and Abidjan (white dots). In Egypt, for instance, diesel subsidies amount to US$0.58 per litre^46^. Eliminating this subsidy in Cairo would result in annual savings of US$8.6k for owner-operators, compared to US$1.6k with current subsidies. Over a 15-year vehicle lifespan, ending this subsidy could lead to public savings of US$129k per vehicle. A portion of these savings could be reallocated to further reduce the purchase price of electric vehicles or to support the financing of local energy infrastructure upgrades.

### Changes in air pollution exposure

Electrifying minibus taxis would also decrease exposure to traffic-related air pollution (TRAP) due to lower exhaust emissions^47^, preventing numerous diseases and deaths^5,6,48^. Concerns about TRAP originating from minibuses are particularly high due to the prevalence of old second-hand vehicles. In this section, we assess the population’s exposure to TRAP emitted by minibuses by combining the operational minibus fleet simulation with local population densities at a hectare-level resolution. For the nine cities, we estimate the number of people exposed to TRAP, calculate an exposure index to assess the level of exposure, and identify local exposure hotspots where electrification would yield the most important benefits (see “Methods” for methodological details). It should be noted that our TRAP exposure metric is a proxy indicator based solely on traffic volume and distance decay ^49,50^. It does not represent absolute pollutant concentrations, which would require complex dispersion modelling and additional local parameters. Consequently, our results should be interpreted as estimates of potential exposure and associated spatial patterns, rather than as absolute air-quality levels or direct health impacts.

Figure 4a presents, for each city, both the total population and the corresponding share of inhabitants exposed to minibus-related air pollution. We define exposure as living within a 300 m buffer of one or more paratransit routes. This distance delimits an area within which concentrations of traffic-related pollutants remain significantly higher than background levels^51^, capturing the zone of highest exposure to near-road emissions^51^. In general, we found that in all the studied cities, a considerable population is exposed to TRAP. The exposed population is notably high in Alexandria (4 million), Cairo (3.8 million), and Nairobi (3.5 million). Aggregating results across all cities reveals that approximately 23 million people are exposed to TRAP emitted by minibuses. In relative terms, over 50 % of the population lives within the buffer zone in more than half of the studied cities (Nairobi, Freetown, Accra, Alexandria, Bamako). Alexandria exhibits the highest proportion of exposed residents at 63 %, whereas Cairo presents the lowest proportion at 16 %. This lower share reflects Cairo’s large geographical extent and comparatively limited paratransit network coverage. However, because of the city’s large population, the absolute number of residents living within the buffer zone in Cairo (3.8 million) remains among the highest across the nine cities.

Figure 4a also categorises exposure levels as “high”, “medium”, or “low” based on specific thresholds of a TRAP exposure index, which quantifies exposure considering minibus traffic volume and proximity to paratransit roads (see “Methods” for the TRAP exposure index definition). The “high” exposure corresponds to a level of exposure equivalent to that experienced by an individual located at zero distance from a minibus travelling 300 km. “Medium” exposure reflects the exposure from a minibus travelling 150 km, while “low” exposure corresponds to that from a minibus travelling 1 km (i.e., a non-zero exposure). According to this classification, Alexandria again stands out as it has the highest proportion of its population in the “high” exposure category, with 14.1 % of residents experiencing elevated levels of TRAP. Kampala follows with 9.2 % of its population exposed at this level, and Accra with 7.5 %. The results also reveal that all studied cities have a proportion of their population experiencing high exposure levels to TRAP.

Our model also facilitates the identification of TRAP exposure hotspots (i.e. areas characterised by both high TRAP exposure and large population density) thereby pinpointing regions where paratransit electrification would yield the greatest impact. This is accomplished through the calculation of a population-weighted TRAP exposure index. Figure 4c illustrates the spatial distribution of this index for Abidjan. As anticipated, the main exposure hotspots are concentrated near the city centre, where the TRAP exposure index is elevated due to the high volume of paratransit traffic. However, our findings also reveal that exposure hotspots do not invariably coincide with high TRAP exposure areas. In Abidjan, for example, additional hotspots are located in peripheral areas, such as the densely populated Marcory district in the southern part of the city, suggesting that minibus electrification efforts could be beneficial in urban outskirts as well. Conversely, as seen in Cairo (see Supplementary Fig. 2), a high TRAP exposure is sometimes found in areas with a low population density, resulting in only limited hotspots and a small portion of the population being exposed to elevated TRAP levels. This finding underscores the importance of considering both TRAP exposure and population distribution when formulating electrification strategies.

## Discussion

The paratransit sector in Africa is set to undergo considerable changes in the coming years, particularly with the electrification of the current fleet of ageing and polluting minibus taxis in urban areas. In anticipation of this shift, it is crucial to quantify the major implications of electrification to better understand the potential benefits and risks across various locations. Assessing these implications will also help in identifying optimal transition pathways and measures for electrification, including transport policies and energy infrastructure planning.

Our analysis reveals five key findings. Firstly, from an energy perspective, the charging demand resulting from minibus electrification varies considerably across cities. It represents less than 1 % of the current city electricity consumption for Cairo, Alexandria, and Abidjan, but exceeds 20 % for Freetown and Harare, highlighting the need for city-specific infrastructure planning. Secondly, the daily charging needs per vehicle also exhibit substantial variations, ranging from 37.9 to 209.5 kWh on average. This variation stems from differences in minibus operational patterns, emphasising the importance of tailored charging strategies and battery capacities for each location. Additionally, cities like Nairobi and Bamako show tight distributions of vehicle charging needs, whereas Cairo, Harare, and Alexandria display greater variability. While the former could benefit from uniform battery capacities and charging strategies, the latter may require more diverse solutions. Thirdly, from an environmental point of view, transitioning to electric vehicles could prevent the emission of 4.3 to 19.2 tCO$$_2$$/year per taxi (5.9-32.5 tCO$$_2$$/year with a 100 % decarbonised electricity mix). The latter reduction is achieved for Nairobi, due to a high VKT and a low carbon intensity of electricity. Overall, this electrification of all taxis could reduce city emissions by 2.8-54.8 ktCO$$_2$$/year (6.0-66.8 ktCO$$_2$$/year with a 100 % decarbonised electricity mix). Fourthly, the transition to electric minibuses would reduce exposure to local air pollution. Our analysis shows that approximately 23 million people live within a 300 m buffer zone from paratransit routes, exposing them to TRAP emitted by minibuses. In five of the nine studied cities, over 50 % of the population is exposed to these emissions. In addition, a population-weighted exposure index underscores the importance of considering population distribution when assessing air pollution impacts. Our results demonstrate that exposure hotspots - areas with both high TRAP exposure and large populations - do not always coincide with areas of high TRAP exposure. Lastly, the environmental advantages of electrification align with potential economic savings for minibus owner-operators. Annual fuel savings range from US$1.2k for Alexandria to US$14.0k for Harare. In some cities, the savings could be even higher if diesel subsidies were cut. More generally, our findings underscore the complex interplay of factors involved in minibus electrification across different urban contexts, emphasising the necessity of understanding city-specific characteristics and implications to develop tailored solutions.

The concurrent examination of various implications also reveals similarities among certain cities, particularly when considering energy and economic factors, both of which are likely to play pivotal roles in electric minibus adoption. For instance, Cairo and Alexandria both exhibit a low additional electricity demand (<1%) for charging. This suggests their existing power grids could potentially accommodate electric minibuses without substantial infrastructure upgrades. However, these cities face a high per-vehicle charging demand (>150 kWh on average) coupled with modest economic savings of less than US$3.0k per year. This may hinder the adoption of electric minibuses by owner-operators due to the higher battery capacity (and therefore higher upfront cost) that may not be rapidly offset by fuel savings. In contrast, Kampala and Freetown have total additional electricity demands above 20 % but could experience quicker adoption of electric minibuses. In these cities, existing small electric minibuses^38,39,44^, available at comparable prices to traditional diesel minibuses, are likely to meet daily charging needs (<100 kWh on average). Future research should focus on identifying the most suitable electric minibus models for each city to better quantify associated costs and benefits. Such analysis should also take into account the possible long-term evolution of the local transport landscape, as transport authorities could encourage the development of other sustainable modes of passenger transport, such as bus rapid transit systems^52^ or large public buses^36^, in addition to minibuses.

Accommodating a 100 % electric minibus fleet will require careful planning of the energy infrastructure. Finding the optimal solution will also depend on the charging scenario, defining how the charging load will be distributed throughout the day. Given the fragility of many African grids^2,53^, it is crucial to avoid scenarios where all vehicles charge simultaneously at a depot during peak hours, i.e., periods when the electricity demand is the highest. Such a situation not only strains the grid but can also raise the utility’s marginal cost of supply^54^. In countries already facing supply shortages, peak demand may even be met by expensive and carbon-intensive diesel generation^55^, reducing utility revenues while increasing operating costs for minibus owner-operators. High simultaneity in vehicle charging further limits opportunities to share the charging infrastructure, thereby increasing the number of charging stations required. By examining the fluctuation of the proportion of minibuses on the road along the day (see Supplementary Fig. 3), our work also provides insights into infrastructure-friendly charging strategies. For instance, in Nairobi, nearly 50 % of all vehicles are inactive between 9 am and 3 pm, a pattern similarly seen in Harare and Freetown. Moreover, since a large number of taxis have relatively low charging needs, some vehicles may adjust their service schedules from one day to another to charge more frequently outside peak hours. Encouraging such scheduling adjustments could be facilitated through appropriate electricity tariffs at charging stations, which is known to influence the charging behaviour^56^. If vehicles are charged during the day, the charging needs could also be effectively met with locally installed PV systems, therefore mitigating the strains on the electricity grid. In Freetown, about 5 hectares of PV panels (approximately equivalent to a 10 MWp power plant) could meet the average daily charging needs for the city’s vehicle fleet (estimation from PVGIS^57^, assuming 20 % efficient free-standing silicon-based panels with 14 % system loss and optimised slope angles). This PV-based charging approach would not only help to smooth out the charging demand but also to reduce CO$_2$ emissions, given that PV has a much lower carbon intensity (expected to be less than 30 gCO$_2$/kWh due to the high solar irradiance across Africa^58,59^) than grid electricity in the studied countries. The levelised cost of electricity (LCOE) from PV-generated electricity^10^ is also lower than household electricity prices^42^ in many African countries, suggesting that solar power could be a cost-effective option for charging.

We acknowledge several limitations in our study, primarily due to data availability constraints. The main limitations arise from the available GTFS data for African cities^35^. Firstly, due to limited documentation, it is difficult to verify whether the different datasets accurately capture the entire paratransit system in each location. Consequently, the electricity demand implications and environmental benefits at the city level could be underestimated in certain cities. Comparing our findings with existing statistics is also difficult, as many of the existing minibus taxi data is outdated or lacks specificity. Furthermore, it remains unclear to what extent the datasets reflect real-world traffic conditions. For instance, if vehicles operate at lower speeds in practice than those specified in the GTFS feeds, our estimates of VKT and the number of vehicles could be affected. Several datasets also required preprocessing - such as removing non-minibus agencies or excluding weekend services - which could introduce additional sources of bias. While a quantification of potential biases would require detailed, city-specific data that is currently unavailable, we expect the influence of preprocessing-related uncertainties to be small (see the Methods and Supplementary Table 2 for a city-level diagnosis of the potential bias induced by the preprocessing steps).

In addition, due to the unavailability of city-specific figures, estimates such as additional electricity demand for charging or CO$_2$ savings rely on average country-level data. This introduces uncertainty in our results, as uncertainty in the underlying inputs propagates directly through the calculations. To characterise this, we conducted a first-order uncertainty analysis on the key parameters governing charging demand, CO$_2$ savings, and diesel savings (Supplementary Table 4). This analysis captures plausible inter-city variability under current data constraints and therefore provides an upper envelope of expected uncertainty. The largest contribution arises from the assumed variation in the vehicle per kilometre energy use. When combined with the uncertainty in charging efficiency and assuming first-order linear propagation, this yields an uncertainty of approximately 20% in the charging demand. Under the same assumption of linear error propagation, the resulting uncertainty in additional electricity demand for charging per city, diesel savings, and CO$_2$ savings is expected to fall within 30–35%. Importantly, with access to more detailed, city-specific data in future studies, these uncertainties could be substantially reduced. For CO$_2$ savings specifically, note that the uncertainty mainly reflects our use of average rather than marginal emissions factors. This approximation is supported by the generation mixes of the countries included in the study, which are dominated by non-intermittent sources such as hydropower and fossil fuels^41^. Correspondingly, CO$_2$ intensity data show limited temporal variability in most cases^60^. Examining the hourly coefficient of variation of CO$_2$ intensity for 2024^60^, we find values between 3–7 % across all countries except Sierra Leone. The latter shows higher variability (29 %) due to daytime solar production. While a more precise assessment, which would require time-resolved carbon intensity and explicit charging scenarios, is beyond the scope of this study, our findings underscore the need for such analyses in the future, as the share of renewables continues to increase.

As for economic benefits, another source of uncertainty, though difficult to quantify, relates to electricity subsidies. While explicit subsidies (e.g., social tariffs) are generally small^61^, electricity is often indirectly subsidized in African countries through so-called underpricing, as current tariffs do not allow utilities to fully recover their costs. However, much of this underpricing is due to governance and management inefficiencies; if these were reduced to benchmark levels, many utilities would already be close to cost recovery at current tariff levels^61^. For the remaining countries, a sensitivity analysis assuming a 24% tariff increase (the African median underpricing reported in^61^) shows that cost savings remain positive in all cities, even when diesel subsidies are maintained. The least favorable case is Bamako, where savings decrease from US$0.037/km to US$0.015/km, but still remain positive. We also note that the 24% estimate (from 2015 data as no more recent source was available) likely represents a pessimistic upper bound. Many countries have undertaken tariffs reforms in recent years, partly to reducing underpricing^62^. In addition, the growing reliance on low-cost renewable generation is expected to further limit the need for future tariff increases.

The modelling methodology may also introduce certain caveats. As pointed out by Rix et al.^24^, GTFS data does not encompass vehicle movements outside operating hours (e.g., trips to and from parking locations). This omission excludes part of the daily VKT, potentially leading to a slight underestimation of the charging demand. Another limitation is our assumption that each vehicle travels on a single route throughout the day, whereas taxis are known to have a demand-responsive behaviour in some locations^1^. This should be considered when interpreting the charging demand per vehicle and per trip, as illustrated in Fig. 2. Nevertheless, it is likely that this assumption has little effect on the resulting statistical properties, such as the average and standard deviation of the charging needs per vehicle. Should data become available, our model could also be refined to account for local particularities, including factors such as vehicle idling time or switching between different routes. A further limitation arises from our assumption of a fixed per kilometre energy consumption rate. In cities with high per-vehicle charging needs, substantially larger batteries than those typically installed in small electric minibuses could be required, which in turn would increase vehicle mass and thus raise the energy consumption rate^27^. The required battery capacity, however, depends strongly on the adopted charging strategy (e.g., a single daily charging event versus frequent opportunity charging)^63^. These interactions between battery size, vehicle mass, energy consumption, and charging strategy lie beyond the scope of the present work but could be incorporated into a future decision-support tool to aid practical planning of minibus electrification. Lastly, expanding the model to capture the spatial dependencies of EV-PV complementarity at the individual charging station level could provide practical guidelines for charging station operators and infrastructure planners, aiding in the development of PV-based charging stations.

In conclusion, we present a model that leverages GTFS data to assess the main energy, economic, and environmental implications of minibus taxi electrification across nine diverse African cities. Our analysis reveals significant differences in outcomes among these cities, yet consistently demonstrates substantial reductions in CO$_2$ emissions and fuel savings, underscoring its potential as a critical step toward sustainable urban transportation in Africa. However, the increased electrical demand resulting from taxi electrification, especially in cities like Freetown and Kampala, necessitates careful consideration in local energy planning. Our results also uncover variations in charging needs per vehicle, which imply different battery capacity requirements and charging patterns across cities. Interestingly, larger charging needs - likely associated with higher purchase cost for minibuses due to larger battery sizes - do not always correlate with greater fuel savings for owner-operators, potentially hindering the adoption of electric vehicles. Addressing these challenges requires the development of targeted policies, incentives, or smart charging strategies. At the same time, the feasibility of large-scale electrification will depend on enabling conditions such as grid reliability, charging infrastructure expansion, and the capacity of minibus operators to renew their vehicle fleets. Recent progress, especially in East Africa^64^, shows that electric transport can grow quickly when the right conditions are met. More and more projects are proving that electric transport can be scaled up across the continent. Although these efforts are still in their early stages, they highlight both the practical challenges specific to each country and the key factors that help support the successful expansion of electric mobility in Africa. Against this backdrop, our study also offers a framework to guide such measures by evaluating both the energy requirements and the operational characteristics of the paratransit system, such as vehicle availability over time. Future research could build upon this approach by developing city-specific scenarios that account for local infrastructure and energy supply constraints. Moreover, our model could be adapted to analyse the electrification of other forms of public transportation, given the availability of GTFS data, offering a versatile tool for advancing sustainable mobility in other urban contexts.

## Methods

### Model overview

The presented model simulates the operation of a minibus taxi fleet based on GTFS data and quantifies the main energy, economic and environmental implications associated with their electrification. From a general perspective, the methodology comprises three stages (see Supplementary Fig. 1 for a detailed modelling schematic):

1. Preprocessing of GTFS data. This preliminary step aims to ensure a consistent and comparable dataset across the different cities, representing the minibus taxi fleet operation on a typical weekday.
2. Simulation of the minibus fleet operation and energy use. According to the preprocessed GTFS data, the spatial and temporal evolution of the various taxis along their trips are calculated. This step also enables us to examine various operational metrics, including the number of vehicles in operation, vehicle kilometres travelled, and vehicle-kilometres. Simultaneously, at this stage, the electric power dissipated and electric energy use of electric minibus taxis are calculated.
3. Assessment of the electricity demand (daily charging needs and comparison with current city electricity consumption), environmental (changes in CO$_2$ emissions, reduction in traffic-related air pollution exposure) and economic (minibus owner-operator savings) implications. This is realised using the results of the previous step, as well as other context-specific data.

The following sections provide a more detailed description of the three steps, each grounded on different input data and modelling assumptions It should be noted that the modelling results can be easily updated with new inputs as they become available.

### GTFS datasets: data source and preprocessing

The nine GTFS datasets used in our study are all open-source data, made available on GitLab through the DigitalTransport4Africa initiative^35^. All data was downloaded on the same day from the online repository. For reproducibility concerns, the git commit token associated with these various datasets and other information can be found in Supplementary Table 1. Note that some GTFS datasets were not retained for this study, as they did not contain all the information required to simulate minibus operation. In fact, GTFS data comprises several files, of which the following are necessary for our simulation: agency.txt, trips.txt, routes.txt, stops.txt, stop_times.txt, calendar.txt, frequencies.txt, shapes.txt.

Most of the datasets required preprocessing. This process involved three main steps:

1. Removing agencies operating vehicles not relevant to our study (e.g., Abidjan’s datasets included data for *woro-woro* taxis, which are cars, not minibuses)
2. Retaining only weekday trips
3. Eliminating inconsistently linked features (trips, shapes, etc.) to obtain a dataset with a complete relational structure.

In total, six out of the nine datasets have been preprocessed. For detailed characteristics of the GTFS data before and after preprocessing, please refer to the Supplementary Information. Note also that we restrict the analysis to weekdays for two reasons: (1) only Harare and Freetown provide distinct weekday and weekend service patterns, suggesting that weekend variability is not consistently captured across the remaining datasets; and (2) weekday operations generally represent the highest-demand conditions^65^ (as observed in the Harare and Freetown GTFS data), making them the most relevant basis for estimating operational metrics, daily electricity demand for charging, and discussing future requirements for minibus electrification (e.g., required battery capacities).

For Kampala and Cairo, we also implemented a trip combination process to address standardisation issues. In these cities, each change in operating frequency within a single trip was erroneously recorded as a new entry in the trips.txt file, rather than being only logged in the frequencies.txt file. This resulted in multiple fragmented entries for what should have been single trips with varying frequencies. We combined the duplicated entries in the trips.txt file, aligning the data structure with other cities and improving overall consistency in our analysis.

Because preprocessing may influence the representativeness of the weekday GTFS data, we also compiled a GTFS preprocessing diagnosis for each city (Supplementary Table 2), summarising the issues addressed and the estimated risk and direction of any resulting bias. Across cities, the expected risk of preprocessing-related bias is none to small. The only notable source of potential bias arises in Cairo and Kampala, where trip fragmentation may slightly underestimate per-vehicle service frequency and VKT, leading to conservative estimates of charging demand and other per-vehicle metrics. These issues do not affect FKT, which does not depend on reconstructing full vehicle-level trips.

Note that our methodology also allows for the snapping of spatial data from the GTFS files to the existing road network available on OpenStreetMap using the OSMnx package^66^ (see Supplementary Fig. 1). However, we excluded this procedure from the current study due to minimal observed differences for the cities under examination.

### Minibus fleet operation and energy use

Evaluating the minibus fleet operation requires modelling the spatial and temporal evolution of the vehicles during their journey. Our approach is based exclusively on the preprocessed GTFS data along with a minimal set of modelling assumptions, ensuring a fast and easy-to-understand simulation. The methodology is similar to that described by Falchetta et al.^17^, but also evaluates operation metrics at the individual vehicle level (e.g., VKT) in addition to those of the entire fleet (e.g., FKT). For instance, when applied to Nairobi (where the preprocessed GTFS data is identical to the one used in their work), both methods result in an identical FKT value (relative difference of less than 0.2%).

The first aim is to evaluate the movement of a single vehicle along a given trip. In this study, a trip refers to a single run of a minibus taxi along a predefined GTFS route, from its first stop to its last stop, as represented in the trips.txt and stop_times.txt files. This corresponds to the GTFS definition of a trip and should not be confused with the continuous service that a minibus may perform throughout the day in real life. Evaluating the vehicle movement is achieved by dividing the trip into segments between stops (as specified in the stop_times.txt file) and determining the length of these segments using the spatial data available in the stops.txt and shapes.txt files. Adjusting stop locations if needed ensures accurate positioning along the trip path described in the shapes.txt file. With this approach, the segment of the trip a vehicle is travelling through after a certain time following departure can be tracked. It also enables the monitoring of the duration and distance travelled within the segment.

Simultaneously, for each trip, the number of taxis and their spatial distribution are estimated. This is done by populating each trip with the average number of vehicles required to match the headway time specified in the frequencies.txt file. Furthermore, it is assumed that all vehicles are evenly distributed along the trip during service hours since GTFS data only reflects a steady-state regime of operation (i.e., transient aspects such as changes in the number of vehicles at the beginning or end of service hours are not captured). Although these transient aspects could cause some variability in the number of vehicles on the road and, consequently, in the average distance they travel, they are expected to have minimal impact on the results, as significant changes in the number of vehicles in service should be reflected in the frequencies.txt file. Knowing both the number of vehicles in operation and the evolution of a given vehicle along each trip, trip-specific metrics such as the FKT can easily be calculated. Similarly, considering independent trips (i.e., assuming taxis only operate on a single trip), estimates such as the VKT for individual vehicles can be evaluated. This assumption is justified by our focus on understanding the statistical characteristics of the overall fleet rather than examining the detailed journey of specific vehicles.

Electric energy use is estimated based on a per kilometre consumption rate of 0.39 kWh/km, a value derived from electro-kinetic simulation based on the characteristics of a typical 16-seater minibus^27^. This figure is slightly higher than expected from manufacturer data^38,39,67^ or other independent estimates^68^ because it reflects the energy consumption in real operating conditions, notably taking aggressive driving patterns into account. As for the power dissipated, it is estimated by dividing the energy use by the travel time of each segment, assuming a constant power dissipation during the vehicle movement between stops. Note that the consumption rate of 0.39 kWh/km is applied uniformly across all cities, as route-specific factors such as route topography or driving behaviour are expected to have only a small effect on the average per-vehicle or fleet-wide results^69^, while incorporating them would add substantial modelling complexity.

### Assessment of electricity demand, CO$_2$ and economic metrics

When estimating charging needs based on energy consumption, we apply a charging efficiency of 90 %^70^ as our analysis primarily focuses on the electricity needed to be drawn from the grid. Additionally, we consider 250 working days for assessing annual charging needs. To compare those needs with the current city electricity consumption, the city population and current per capita electricity consumption of each city are estimated. The city population is determined using the GHS-POP 2020 population distribution dataset^37^, considering only inhabitants living within the bounding box surrounding the paratransit routes. This procedure aims to enable a fair comparison between cities by accounting for geographical area differences. As for the current electricity consumption per capita, it is estimated using recent averages at the country level^71^.

CO$_2$ emission reductions are calculated by comparing diesel and electric vehicle emissions. For diesel emissions, a carbon intensity of 2.7 kgCO$_2$/l and a fuel consumption of 0.1 l/km are chosen based on the average values for the diesel counterpart of the aforementioned 16-seater minibus^72^. It should be noted that this is a conservative estimate since many minibuses in operation are likely to emit more CO$_2$ due to their age and higher capacity^73^. With regard to CO$_2$ emissions for electric vehicles, the carbon intensity of the local electricity generation is used^41^.

The cost savings of operating an electric minibus are determined by comparing diesel prices with household electricity tariffs^42^. Household tariffs are used to reflect the financial perspective of minibus owner–operators. The marginal cost to utilities of supplying additional electricity, which may vary over time even though customers typically not face time-of-use pricing in Africa^62^, is beyond the scope of this analysis. The cost per litre of diesel is evaluated with and without direct fuel subsidies^46^, enabling an assessment of potential economic benefits should these subsidies be removed. Indirect subsidies, such as environmental externalities or foregone tax revenues, are not taken into account in our calculation. It should also be noted that 2022 subsidy data are used, as only projections are available beyond this time frame.

National averages from harmonised international datasets are used for electricity consumption per capita^71^, grid carbon intensity ^41^, and electricity prices^42^. These datasets apply consistent definitions and methodologies across countries; therefore, no further standardisation was required. Using national averages introduce uncertainty for cities whose electricity supply characteristics or tariff differ from the national average, but this effect is expected to be small, as in most African countries electricity grids are centrally operated and managed by central public utilities^2,74^. Moreover, the modelling framework can readily incorporate city-specific data as it becomes available.

### Traffic-related air pollution exposure modelling

The exposure to TRAP emitted by minibus taxis is indirectly assessed using a distance-weighted traffic volume indicator. Our approach is similar to other work^49,50^, using the daily FKT as a metric for traffic volume and an exponential decay for distance weighting. For each pixel *k*, the local TRAP exposure indicator $I_k$ is calculated as follows

1 $$\begin{aligned} & I_k = \sum _{g\ \in D_k} \epsilon _g \cdot f(d_{gk}) \end{aligned}$$

2 $$\begin{aligned} & \epsilon _g = \sum _{i\ \in g} \delta \textrm{FKT}_i \end{aligned}$$

3 $$\begin{aligned} & f(d_{gk}) = e^{-\lambda d_{gk}} \end{aligned}$$

with *g* the index of a pixel located within the buffer zone $D_k$ (a circle centred on *k* with a radius of 300 m), $\epsilon _g$ an emission index of pixel *g*, and $f(d_{ik})$ a distance weighting function. The 300 m buffer distance is widely adopted measurement in air pollution studies^51^, effectively capturing the area of highest exposure to near-road emissions. Within this distance, pollutant concentration levels decrease rapidly, after which the rate of decay noticeably diminishes. The value of $\epsilon _g$ reflects the local daily traffic volume (proportional to the total pollutant emissions) by considering the fraction of vehicle-kilometres $\delta \textrm{FKT}_i$ associated with each trip *i* that is located within the boundaries of pixel *g*. The function $f(d_{gk})$ represents the dispersion of pollutants with distance from the paratransit roads. An exponential decay model is selected, which represents the well-documented phenomenon of pollutant concentration decreasing near roads^51,75^.

The decay rate $\lambda$ could vary depending on the type of pollutant and the local environment. To avoid overestimating TRAP exposure, a high decay rate of 0.0064 m$^{-1}$ is selected^51^. In addition, it should be noted that the distance $d_{gk}$ aims to represent the average distance between the population of pixel *k* and the road segments of pixel *g*. However, as we do not have information about population distribution within a given pixel, for $g \ne k$, this distance is set equal to the distance between the two pixel centres. However, for $g = k$, this assumption does not hold true - in such cases, a distance equal to the average distance between two randomly distributed points within the pixel is chosen.

Note that $I_k$ has the dimension of a distance because the function *f* is dimensionless. As a first approximation, it represents the level of exposure equivalent to that experienced by an observer situated close to a vehicle travelling a distance of $I_k$ kilometres. For the cities under consideration, the maximum exposure index varies between 721 km (Abidjan) and 1213 km (Kampala). To simplify, the exposure level has been condensed into 3 categories (“high”, “medium”, “low”) as previously outlined in the results section. The “high” level threshold is set at an exposure index value of 300 km. This is equivalent to the cumulative exposure an individual would experience from the nearby emissions of a minibus taxi travelling that distance. The “medium” level is set at an exposure index value of 150 km, while the “low” level corresponds to an exposure value of 1 km. For understanding the magnitude of the exposure in real-world scenarios, the “high” exposure threshold is also approximately equivalent to the exposure index calculated at the centre of a straight busy road, with a vehicle frequency (1/$\tau$) of one vehicle per second over an exposure time *T* of 15 minutes

4 $$\begin{aligned} I_\infty = 2 \cdot \int _0^{+\infty } \frac{d\textrm{FKT}}{dx} e^{-\lambda x} dx = 2 \cdot \int _0^{+\infty } \frac{T}{\tau } e^{-\lambda x} dx = \frac{2 \cdot T}{\tau \cdot \lambda } \simeq 280~km \end{aligned}$$

with *x* being the distance from the centre to the road segment of length *dx*.

After calculating the indicator $I_k$ for each pixel, the local population exposure is derived using the GHS-POP population density dataset^37^. Ultimately, the population-weighted TRAP exposure is determined by multiplying the TRAP index map with the exposure map and then multiplying by GHS-POP population density.

## Supplementary Information

Supplementary Information.

## Data Availability

All figures and associated raw data are publicly available from https://doi.org/10.5281/zenodo.13682765. This repository also contains the GTFS data which were used in the simulations for the cities under study. Additional input data for estimating population, electricity demand, CO_2_ emissions, TRAP exposure, and economic savings were obtained from publicly available sources, as detailed in the Methods section.

## Code availability

The commented source code used to conduct model simulations in this work is available from https://doi.org/10.5281/zenodo.13683085. This archive also contains the necessary data to ensure the complete reproducibility of our analyses.
