# Supplementary material for: Energy, environment, and economy implications of electrifying minibus taxis in African cities
Source: Sci Rep. 2026 Mar 30;16:10661. doi: 10.1038/s41598-026-45790-w (PMC13039481; doi:10.1038/s41598-026-45790-w)
Supplement: Supplementary file 1 — Supplementary Information. [file 41598_2026_45790_MOESM1_ESM.pdf]

Energy, environment, and economy implications of electrifying minibus taxis in African cities

Jérémy Dumoulin[1,*], **Alejandro Pena-Bello**[1], **Noémie Jeannin**[1], **Christophe Ballif**[1], **and Nicolas Wyrsch**[1]

[1]Photovoltaics and thin film electronics laboratory (PV-LAB), École Polytechnique Fédérale de Lausanne (EPFL), Institute of Electrical and Microengineering (IEM), Neuchâtel, Switzerland
[*]jeremy.dumoulin@epfl.ch

ABSTRACT

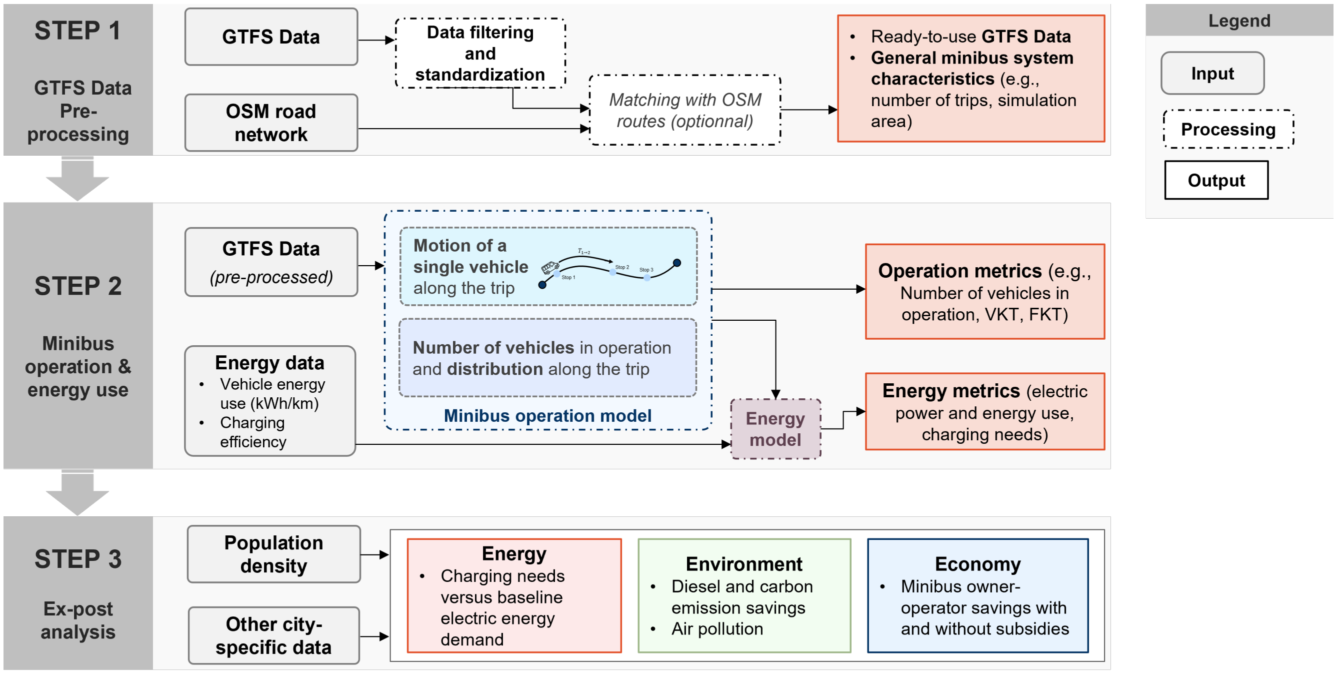

Figure 1. Methodology

Table 1. General attributes of the GTFS feeds describing the minibus taxi systems in cities after preprocessing (any changes from original values are indicated in brackets)

City	Commit token	Agencies	Trips	Routes	Stops	Area (km^2)
Abidjan	da5d9fafc21d75d355ba9 cc41df7a6e1a3734d52	7 (24)	218 (830)	109 (398)	1329 (3820)	1385.1 (1680.5)
Accra	f04f2f1209f4235a832c7 008463bd6eb019e32a2	1	554	277	2525	1299.3
Alexandria	a47464a4a9a3480f200b6e 0aa3d9a85690fc453e	3 (5)	171 (192)	92 (104)	422 (441)	3676.3
Bamako	23aa917cb7c76bafdad62 3a96f1d8e6f2ec136b0	1	210	105	3457	2342.7
Cairo	ae279a3097e0c1aa1f990 03acf0724f0da947778	4 (6)	283 (730)	170 (214)	878 (1302)	5602.6
Freetown	08f84a138e3a3833e5ce7 eac1719e61d89bb6c06	1 (4)	207 (404)	69 (103)	740 (843)	495.8 (572.9)
Harare	178fa08537240b8a3faf8 f2d172186b600fa1244	1	486 (972)	486	2127 (2255)	4859.2
Kampala	42e89cddcbd41d48fa596 04e11fc22b66215a627	1 (2)	551 (4696)	369 (397)	1233 (1242)	1629.3
Nairobi	4a85af50353548f476d18 f60984115c78e20fbcc	1	272	136	4284	2467.3

Table 2. GTFS preprocessing diagnosis for each city.

City	Preprocessing intensity	Issues addressed	Estimated risk and direction of bias
Abidjan	High	Removed 17 non-minibus agencies (formal buses, ferries, conventional car taxis) out of 24 in the original dataset	None–Small (low risk of removing some informal minibus-like services if mislabeled as formal modes)
Accra	None	GTFS feed contains only minibus services	None expected
Alexandria	Low	Removed formal bus agencies (well labeled in the GTFS dataset)	None expected
Bamako	None	GTFS feed contains only minibus services	None expected
Cairo	Medium	Removed formal bus agencies (well labeled); resolved moderate minibus trip fragmentation (multiple trips from the same minibus split across time windows)	Small – potential underestimation of per-vehicle service frequency and VKT (low risk, as trips are well labeled); no influence on FKT
Freetown	Medium	Removed weekend services and non-minibus agencies (well labeled)	None expected
Harare	Small	Removed weekend services	None expected
Kampala	High	Removed formal bus agencies (well labeled); resolved severe minibus trip fragmentation (many trips split across time windows)	Small – potential underestimation of per-vehicle service frequency and VKT (low risk, as trips are well labeled); no influence on FKT
Nairobi	None	GTFS feed contains only minibus services	None expected

Table 3. City-specific data used for calculating CO_2 emission reductions, economic savings, and current city electricity consumption.

City	City population	Per capita electricity consumption (kWh/year)	Per city electricity consumption (MWh/day)	Electricity CO_2 intensity (kgCO_2/kWh)	Electricity price (US$/kWh)	Diesel price (US$/l)	Diesel subsidies (US$/l)
Abidjan	5,696,639	399.6	6236.6	0.411	0.118	1.165	0.1
Accra	4,736,062	637.8	8275.8	0.361	0.122	1.087	0.0
Alexandria	6,429,284	1851.1	32606.2	0.470	0.017	0.209	0.58
Bamako	3,850,818	154.8	1633.2	0.463	0.214	1.304	0.0
Cairo	23,359,844	1851.1	118469.6	0.470	0.017	0.209	0.58
Freetown	1,786,162	24.9	121.85	0.048	0.155	1.524	0.0
Harare	2,715,440	502.7	3739.9	0.392	0.064	1.680	0.0
Kampala	4,397,849	96.0	1156.7	0.052	0.169	1.309	0.0
Nairobi	6,801,083	223.2	4158.9	0.101	0.212	1.385	0.0

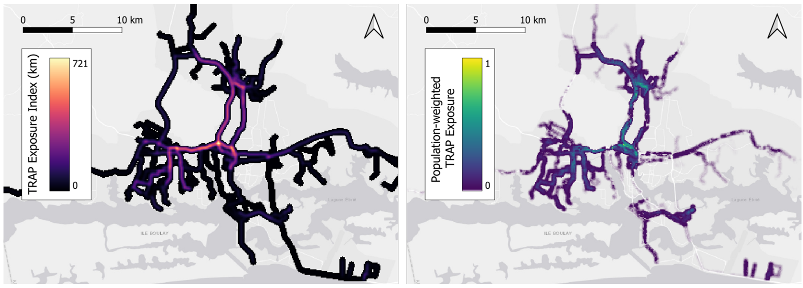

(a) Abidjan

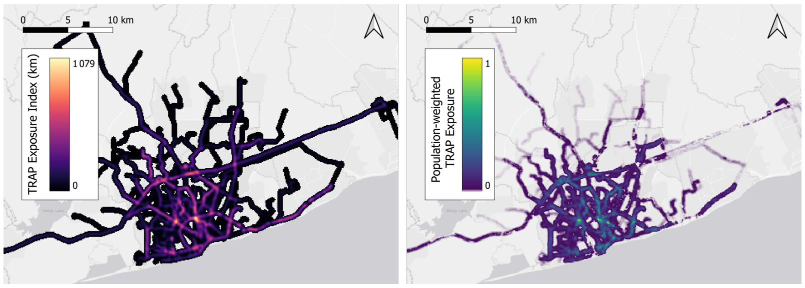

(b) Accra

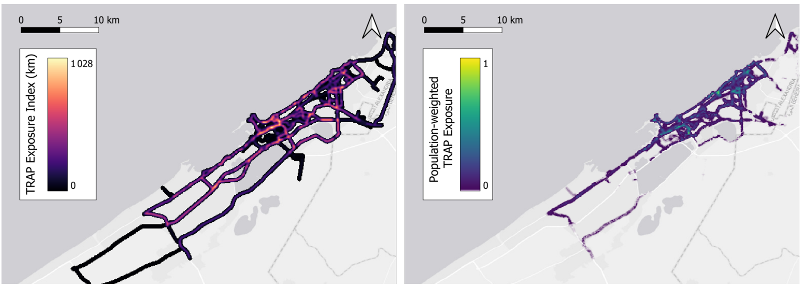

(c) Alexandria

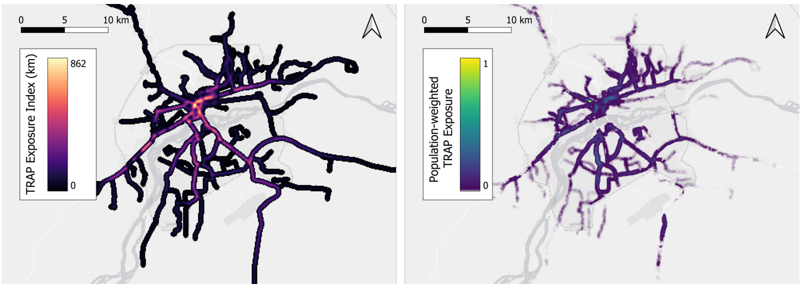

(d) Bamako

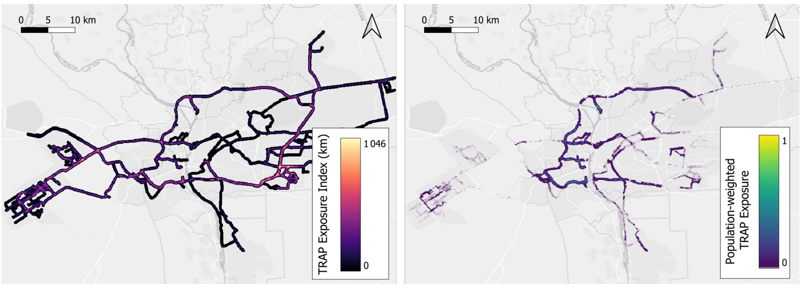

(e) Cairo

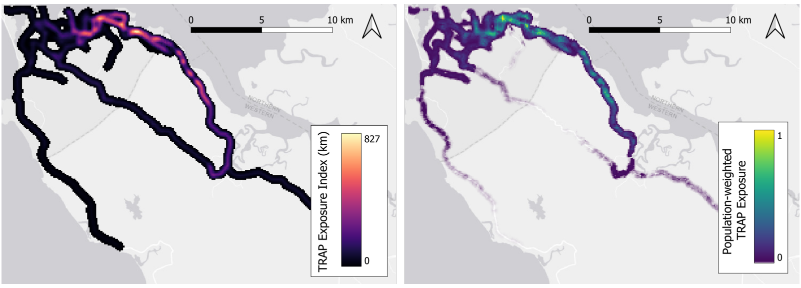

(f) Freetown

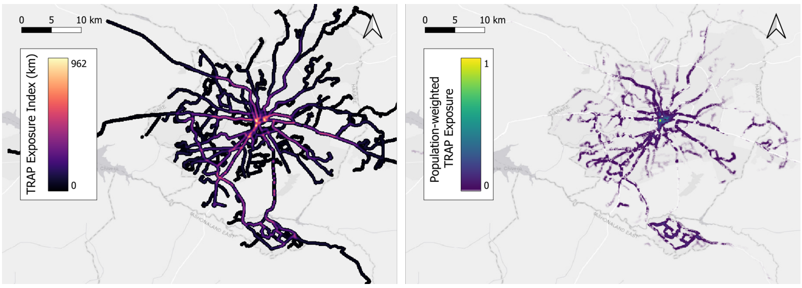

(g) Harare

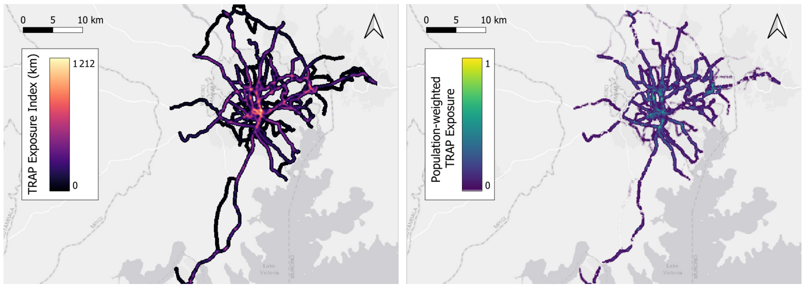

(h) Kampala

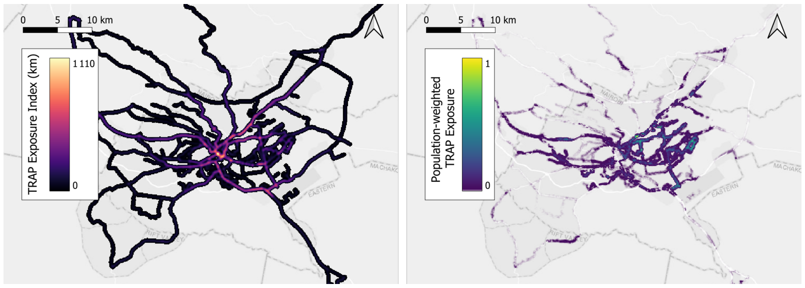

(i) Nairobi

Figure 2. TRAP exposure index map (left) and population-weighted TRAP exposure map (right) for the studied cities. Note that the colour scale of the TRAP exposure index changes from one city to another in order to better see the differences with the population-weighted TRAP exposure index.

Table 4. Uncertain input parameters, affected outputs, and assumed coefficient of variation (CV).

Parameter	Affected output(s)	CV (%)	Reference / justification	Notes
Energy consumption (kWh/km)	Total and per-vehicle charging demand (and related indicators)	13	Standard deviation between recommended energy consumption under different driving conditions (Table 6 of Hull et al.[1])	This CV captures inter-city variations (e.g., hilly cities such as Freetown and Kampala will have higher energy consumption). Therefore, we used the standard deviation across the extreme values[1].
Charging efficiency (-)	Total and per-vehicle charging demand (and related indicators)	5	Standard deviation of the four charging-efficiency values reported in Reick et al.[2].	Captures many losses[2]. Empirical data remain limited, with no studies focused on e-minibus charging systems.
Carbon intensity (gCO_2/kWh)	CO_2 reductions	5	Average hourly coefficient of variation of CO_2 intensity in 2024 for the nine studied countries[3].	Sierra Leone excluded (see the limitations section for a discussion)
Diesel fuel consumption (l/km)	CO_2 reduction ; Diesel savings	10	Own estimate based on a review of variations in publicly available data for a typical 16-seater minibuses[4].	Expected to have smaller variability than electric energy consumption due to relatively stable combustion process and engine efficiency,
Diesel carbon intensity carbon ($kgCO_2/l$)	CO_2 reduction	0.5	Yang et al.[5] (rounded up to 0.5%)	Small uncertainty because diesel emissions are chemically well-defined; and real-world variation is minimal[5].
City population (-)	Relative per-city electricity demand for charging	5	Own estimate. The primary issue in GHS-POP is the misallocation of population within some cities rather than aggregate estimates that depend on census data[6]	Affects the denominator of the charging demand relative to the current city electricity consumption (no influence on the per-vehicle charging demand, CO_2, or diesel savings).
Per-capita electricity consumption (kWh/person/year)	Relative per-city electricity demand for charging	10	Own estimate. National per-capita electricity consumption mixes low-consuming rural populations with higher-consuming urban populations.	Affects the denominator of the charging demand relative to the current city electricity consumption (no influence on the per-vehicle charging demand, CO_2, or diesel savings).

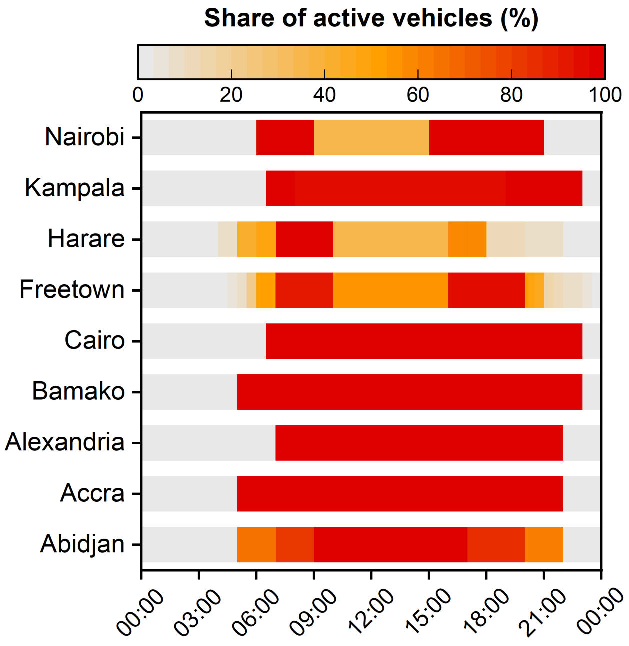

Figure 3. Evolution of the proportion of active minibus taxis throughout the day (number of vehicles on the road out of the total number of vehicles), revealing a different pattern for each city.

References

1. Hull, C., Giliomee, J. H., Collett, K. A., McCulloch, M. D. & Booysen, M. J. High fidelity estimates of paratransit energy consumption from per-second GPS tracking data. *Transportation Research Part D: Transport and Environment* **118**, 103695 (2023).

2. Reick, B., Konzept, A., Kaufmann, A., Stetter, R. & Engelmann, D. Influence of charging losses on energy consumption and co_2 emissions of battery-electric vehicles. *Vehicles* **3**, 736–748 (2021).

3. Electricity Maps. Carbon intensity data (version january 27, 2025). https://www.electricitymaps.com (2025). Accessed: 2025-11-27.

4. Toyota. Toyota Hiace Ses'fikile 2.5D 16s specifications. https://freewaytoyota.co.za/site/wp-content/uploads/2021/01/Sesfikile_Leaflet.pdf (2024). [Online: accessed 06-05-2024].

5. Yang, J. *et al.* Real-world emission characteristics and driving factors of diesel trucks : Insights from plume chasing experiments. *Atmospheric Environment : X* **25** (2025).

6. Kuffer, M., Owusu, M., Oliveira, L., Sliuzas, R. & van Rijn, F. The missing millions in maps: Exploring causes of uncertainties in global gridded population datasets. *ISPRS International Journal of Geo-Information* **11** (2022). URL https://www.mdpi.com/2220-9964/11/7/403.
